# Medical News

**Published:** 1856-04

**Authors:** 


### THE MEDICAL EXAMINER.

PHILADELPHIA, APRIL, 1856.

### MEDICAL NEWS.

American Medical Association.—The Ninth Annual Meeting of the American Medical Association will be held in the City of Detroit, Michigan, on Tuesday, May 6th, 1856.

The secretaries of all societies and other bodies entitled to representation in the Association, are requested to forward to the undersigned correct lists of their respective delegations, *as soon as they may be appointed*; and it is *earnestly* desired by the Committee of Arrangements, that the appointments be made at as early a period as possible.

The following extracts are from Article 2d of the Constitution:

"Each local society shall have the privilege of sending to the Association one delegate for every ten of its regular resident members, and one for every additional fraction of more than half this number.

"The Faculty of every regularly constituted Medical College or chartered school of medicine, shall have the privilege of sending two delegates. The professional staff of every chartered or municipal hospital, containing a hundred patients or more, shall have the privilege of sending two delegates; and every other permanently organized medical institution, of good standing, shall have the privilege of sending one delegate.

"Delegates, representing the Medical Staff of the United States Army and Navy shall be appointed by the Chiefs of the Army and Navy Medical Bureau. The number of delegates so appointed shall be four from the army medical officers, and an equal number from the navy medical officers."

The latter clause, in relation to delegates from the army and navy, was adopted as an amendment to the Constitution, at the meeting of he Association held in New York, in May, 1853.

*** Medical Journals, &c., please copy.

WILLIAM BRODIE, M. D., Detroit, Mich.,
One of the Secretaries.

We are requested to state that the meeting will be held in Firemen's Hall, corner of Jefferson Avenue and Randolph Streets, where gentlemen will be found ready to give all information relating to the accommodation of members. Similar information may be obtained, also, at Dr. Brodie's Office, No. 135 Jefferson Avenue, Masonic Hall.

---

Appointments.—Dr. James J. Levick has been elected a Physician to the Pennsylvania Hospital. The Medical Board, as now constituted, consists of Drs. Wood, Pepper, Gerhard and Levick; the Surgical, of Drs. Norris, Peace, Neill and Pancoast.

Dr. Alfred T. King, of Greensburg, Penna., has been appointed Professor of Practice, and Dr. George Dock, Professor of Surgery, in the Philadelphia College of Medicine. Dr. Dock is well known to the readers of the Examiner through his various and excellent contributions to its pages.

---

A *mandamus* has been granted by the Supreme Court of the State of Michigan, compelling the Board of Regents of the University to appoint a Professor of Homœopathy, according to the act of the Legislature that created the chair, or else to show cause why the same is not done. We should not imagine it to be very difficult to meet the last alternative.

---

Dr. O. C. Gibbs, formerly of Perry County, Ohio, has lately moved to Frewsburgh, Chatauque County, N. Y. We have not the pleasure of knowing Dr. G., but judging from the articles he has contributed to the Examiner, as well as to other medical journals, we believe him to be a gentleman in every way worthy of the confidence and respect of the public. We wish him success in his new location.

---

We learn from the report of the Chief Resident Physician of the Philadelphia Hospital, Dr. R. K. Smith, that during the last six months 3,019 patients were under medical treatment, of whom 2,077 were discharged cured, 289 relieved, 291 died, and 362 are now in the house. The per centage of deaths for the number treated was a single fraction of a hundred over nine per cent., and in the Lunatic Asylum, where 554 cases additional to the above were under treatment, was but 5 41.100.

---

Dr. Kirkbride's report of the Pennsylvania Hospital for the Insane, for 1855, informs us that the total number of patients in the Hospital, during the year was 399; the highest number at any one time was 242, the lowest, 223. 176 were admitted and 169 discharged.

Of the discharged, 101 were cured; much improved, 13; improved, 23; stationary, 11; died, 21.

A considerable part of the Report is taken up with an earnest appeal to the public for assistance in erecting and endowing a new hospital for the insane, on ground contiguous to the present institution. The need is a great one, and we ardently hope that the small amount required will be soon procured.

---

On the Continent during the past month there does not seem much of novelty. M. Paul Dubois has been inquiring into an endemic of puerperal fever which has been prevalent in Paris, very inopportunely for the accouchement of the Empress Eugenie, which occurs in a few days. From various communications with the French capital, and from meeting various Paris men recently in London, it seems they now understand how to treat those cases better than they did. Dr. Ch. Martius has published a series of sketches recently (in the *Deutsche Klinik,*) of what he has seen in London, and some of the views of Graves to "feed fevers," and of Dr. Todd to treat a whole host of inflammations in the same way, has caused an extraordinary sensation generally through France and Germany. Mr. Skey has been giving some most admirable lectures also in London on chronic abscesses, in a surgical point of view, he believing they have their origin in a state of the system half fever, half inflammation, which he calls "below par," of which he has been giving a series of most brilliant exemplifications. Dr. Todd's erysipelatous poisoning of the blood from fever, Skey's "below par," and Graves's "feeding fever," have all a common origin; so much so that Mr. Skey now tells his class they have learnt too much surgery, and they must give it up for medicine!—*Dublin Med. Press.*

---

The University authorities of Edinburgh have awarded a pension of £250 per annum to Dr. Alison, Emeritus Professor of Medicine. We believe that the Patrons and Senatus have, in this instance, given the largest amount at their disposal. In addition, Dr. Alison's case is deserving the attention of Government, and we trust that proper means will be adopted to have it adequately represented in the right quarter. Eminence in medicine, like eminence in any department of science or art, is entitled to public recognition; but Dr. Alison's labors among the poor, and his important investigations regarding pauperism and epidemics, have placed him in the foremost ranks as a public benefactor, and have established a claim which no government could consistently overlook.—*Edinburgh Med. Journ.*

---

We publish the following circular with much pleasure:

HALL OF THE "AMERICAN MEDICAL SOCIETY IN PARIS,"
*Rue des Quatre Vents, No.* 6, *Paris, July 4th,* 1855.

SIR,—In obedience to a resolution of "The American Medical Society in Paris," the undersigned, in addressing you a copy of its lately published Constitution and Catalogue, have been instructed at the same time to direct your attention to certain facts in regard to the Society, which it is hoped may prove of interest to you and to the profession generally in the United States.

This Society occupies an exceptional position, and one which might enable it to be the medium of doing great service to the profession in the United States, by means of an international exchange of professional labors.

The American physician still encounters in the wards of the Parisian Hospitals the sneering question, "What have you ever done in America to advance the science of Medicine?"

The almost complete forgetfulness with which the profession of the United States is passed over on the one hand, and the ridicule with which it is treated on the other, have taught such of us as know most of the opinions and the tone of the medical men of this country, that a remedy for this state of things is demanded, and that some means ought to be adopted to put the remedy into execution.

There are honorable exceptions to these appreciations of the labors of the profession in the United States, but they do not extend to any considerable portion of the profession of this country; and when we hear so distinguished a man as the learned Professor of Surgery of the Faculty of Medicine of Paris, declare in his official lecture at the School of Medicine, while ridiculing American pretensions to originality in surgery, "that because Americans had discovered Anæsthesia, they had become so puffed up as not to be able longer to realize their true position in the world of Surgery," we think that some efforts ought to be made by the profession in the United States to correct this erroneous estimate of their labors, and to show to the profession of France that their brethren of the United States have rendered valuable services even to French Surgery.

"The American Medical Society in Paris" has, by the facts which it has furnished from time to time to French Medical Authors, contributed in a measure to dissipate these mal-appreciations, and has thus succeeded in introducing into French works flattering notices of the successes of American Surgery—successes of which the eminent French authors had no previous knowledge, and which would never have found a place in their works, but for the existence of the American Library at Paris.

The Committee therefore appeals to the profession in the United States, to authors and publishers especially, to send contributions to the Library of "The American Medical Society in Paris."

The Society places its claims for the sympathy of the profession at large upon national grounds; for from its geographical position and frequent change of members, it is supported more by patriotic than by personal motives, and it is upon these grounds that this appeal is made.

Through the politeness of Mr. Bossange, bookseller, No. 138 Pearl street, New York, to whom all books should be addressed, and the obliging forwarding house of Messrs. Livingston, Wells & Co., No. 8 Place de la Bourse, Paris, all contributions addressed to the Society will arrive safely.

W. E. Johnston, M. D., *Pres't.*,

David P. Holton, M. D. *Libr.*,

Saml. Gourdin, M. D., *Cor. Sec'y.*

} *Committee.*

---

Obituary.—Died, on the 13th of February, Prof. Isaac A. Pennypacker, of the Philadelphia College of Medicine, in the 44th year of his age. Dr. Pennypacker's many excellent qualities render his loss a severe one to all who were acquainted with him.

